# New Insight on the Cytoprotective/Antioxidant Pathway Keap1/Nrf2/HO-1 Modulation by *Ulva intestinalis* Extract and Its Selenium Nanoparticles in Rats with Carrageenan-Induced Paw Edema

**DOI:** 10.3390/md21090459

**Published:** 2023-08-22

**Authors:** May Almukainzi, Thanaa A. El-Masry, Hend Selim, Asmaa Saleh, Mostafa El-Sheekh, Mofida E. M. Makhlof, Maisra M. El-Bouseary

**Affiliations:** 1 Department of Pharmaceutical Sciences, College of Pharmacy, Princess Nourah bint Abdulrahman University, Riyadh 11671, Saudi Arabia; mkalmukainizi@pnu.edu.sa (M.A.); asali@pnu.edu.sa (A.S.); 2 Department of Pharmacology and Toxicology, Faculty of Pharmacy, Tanta University, Tanta 31527, Egypt; 3 Department of Biochemistry, Faculty of Pharmacy, Tanta University, Tanta 31527, Egypt; 4 Botany Department, Faculty of Science, Tanta University, Tanta 31527, Egypt; mostafaelsheikh@science.tanta.edu.eg; 5 Botany and Microbiology Department, Faculty of Science, Damanhour University, Damanhour 22511, Egypt; mofida_makhlof@yahoo.com; 6 Department of Pharmaceutical Microbiology, Faculty of Pharmacy, Tanta University, Tanta 31527, Egypt

**Keywords:** *Ulva intestinalis*, anti-inflammatory, cytoprotective effect, selenium nanoparticles, Keap-1, Nrf2, Egypt

## Abstract

Currently, there is growing interest in exploring natural bioactive compounds with anti-inflammatory potential to overcome the side effects associated with the well-known synthetic chemicals. Algae are a rich source of bioactive molecules with numerous applications in medicine. Herein, the anti-inflammatory effect of *Ulva intestinalis* alone or selenium nanoparticles loaded with *U. intestinalis* (UISeNPs), after being fully characterized analytically, was investigated by a carrageenan-induced inflammation model. The pretreated groups with free *U. intestinalis* extract (III and IV) and the rats pretreated with UISeNPs (groups V and VI) showed significant increases in the gene expression of Keap1, with fold increases of 1.9, 2.27, 2.4, and 3.32, respectively. Similarly, a remarkable increase in the Nrf2 gene expression, with 2.09-, 2.36-, 2.59-, and 3.7-fold increases, was shown in the same groups, respectively. Additionally, the groups III, IV, V, and VI revealed a significantly increased HO-1 gene expression with a fold increase of 1.48, 1.61, 1.87, and 2.84, respectively. Thus, both *U. intestinalis* extract and the UISeNPs boost the expression of the cytoprotective/antioxidant pathway Keap1/Nrf2/HO-1, with the UISeNPs having the upper hand over the free extract. In conclusion, *U. intestinalis* and UISeNPs have proven promising anti-inflammatory activity through mediating different underlying mechanisms.

## 1. Introduction

Inflammation is defined as the body response to various triggers such as certain chemicals, infections, and tissue injury. In this defense mechanism, there are several mediators involved that induce and provoke multiple reactions [1]. Some of the pro-inflammatory mediators are interleukin-6 (IL-6), interleukin-1beta (IL-1β), and tumor necrosis factor-alpha (TNF-α), which are secreted through the inflammatory process to intensify the inflammation process [2,3]. Additionally, some enzymatic pathways are known to be related to augmenting the inflammatory responses, such as cyclooxygenase 2 (COX-2), the key enzyme that produces prostaglandin E2 during inflammation [4,5,6]. Being secreted by the inflammatory cells, reactive oxygen species (ROS) have a crucial role in defense mechanisms and aggravate the process of oxidative stress, hence being considered active contributors to inflammatory reactions [7]. Kelch-like epichlorohydrin-related proteins (Keap1) and multifunctional regulator nuclear factor erythroid 2-related factor (Nrf2) are known as vital modulators of oxidative stress; they provide a cytoprotective effect through inhibiting oxidative stress and inflammatory response. When activated, Keap1 is proven to induce phosphorylation of Nrf2, which translocates to the nucleus [8,9,10]. Subsequently, Nrf2 triggers the production of antioxidant enzymes, including via induction of the Nrf2 pathway, which stimulates the production of heme oxygenase-1 (HO-1) and so suppresses the activation of other inflammatory mediators [11].

It has been stated that finding safe and effective agents that can tackle inflammation is a matter of difficulty, since using conventional nonsteroidal anti-inflammatory drugs comes with several side effects; not to mention the long-term-use-related serious adverse effects imposed on the renal, gastrointestinal, and cardiac systems. Lately, several studies have introduced natural products as a source of several bioactive compounds and as having potential for medical anti-inflammatory actions, with the advantage of being safe, effective, and highly biocompatible [12,13].

Macroalgae, commonly known as seaweeds or sea vegetables, are plant-like organisms that usually grow attached to rocks on coasts. They are commonly categorized by their pigmentation, morphology, anatomy, and nutritional composition as red (*Rhodophyta*), brown (*Phaeophyta*), or green seaweeds (*Chlorophyta*) [14,15,16,17]. In addition to being healthy suppliers of minerals, fiber, and proteins, they also contain balanced necessary amino acids, pigments, and fatty acids, as well as other key metabolites for a variety of novel pharmaceutical and industrial uses, as well as green bionanofactories [18,19,20,21,22].

*Ulva intestinalis* Linnaeus, 1753, formerly identified as *Enteromorpha intestinalis* and classified under the phylum *Chlorophyta*, order *Ulvales*, and genus *Ulva,* represents one of the most widely distributed genera of marine seaweeds worldwide [23,24]. *Ulva intestinalis* is characterized by the irregular arrangement of cells and its growth as a tube of 1–3 cm in width and 60 cm in length [25,26]. In the initial phase of growth, its edge appears smooth and progressively wrinkles during its development. Additionally, the color is linked to the state of growth, changing from dark green to light green or yellowish green [26,27].

*Ulva intestinalis* is full of various health-promoting bioactive components such as polysaccharides, phlorotannins, lectins, fatty acids, terpenoids, alkaloids, steroids, proteins, carbohydrates, and many additional components that give it a promising role as a safe and natural antibacterial, antioxidant, antiviral, anti-inflammatory, and even anticancer agent, and it is worth mentioning the use of *Ulva* extracts to synthesize nanoparticles [21,28,29,30,31].

Nanotechnology has recently opened interesting new avenues for research in the realm of nanomedicine [32]. Numerous nanoparticle types exist, including metallic, non-metallic, carbon, semiconductor, and others. Nevertheless, metallic nanoparticles are more significant and have been crucial in the areas of electronics, cosmetics, and medicine [33,34]. Various chemical and physical techniques have been used to synthesize nanoparticles; however, these techniques are always criticized for being expensive and dangerous. Because of their toxicity and unfriendliness to the environment, various techniques such as laser ablation, sol-gel, chemical reduction, solvothermal, and inert gas condensation should be avoided [35]. Due to its affordability and biosafety, green nanotechnology has recently received a lot of attention in the field of nanoparticle synthesis [36]. Gold, silver, copper oxide, and zinc oxide are the most often biosynthesized NPs out of the many series of algae-mediated synthesis [37]. A few studies have so far documented the use of green seaweed (marine algae) for the synthesis of Se-NPs [38]. According to the literature, selenium nanoparticles (SeNPs) have lower toxicity and better bioavailability than other forms of selenium and are, therefore, more effective at combating free radicals. Selenium nanoparticles decorated with natural bioactive agents have potential for pharmaceutical application [29,39].

Thus, this study was the first on record to evaluate the anti-inflammatory effect of *Ulva intestinalis* alone or loaded on SeNPs in a carrageenan-induced inflammation model and the possible role of Nrf2, Keap1, and HO-1 pathways in mediating such an effect.

## 2. Results

### 2.1. FT-IR

**Figure 1:**
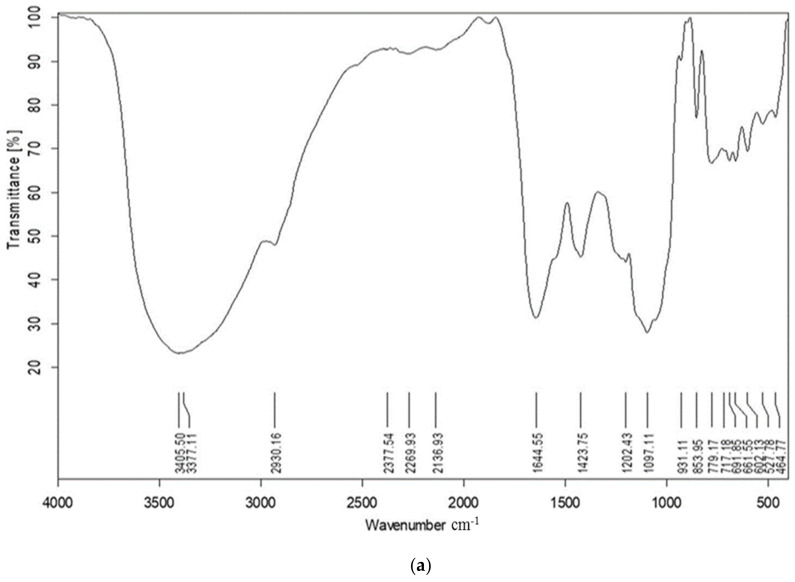

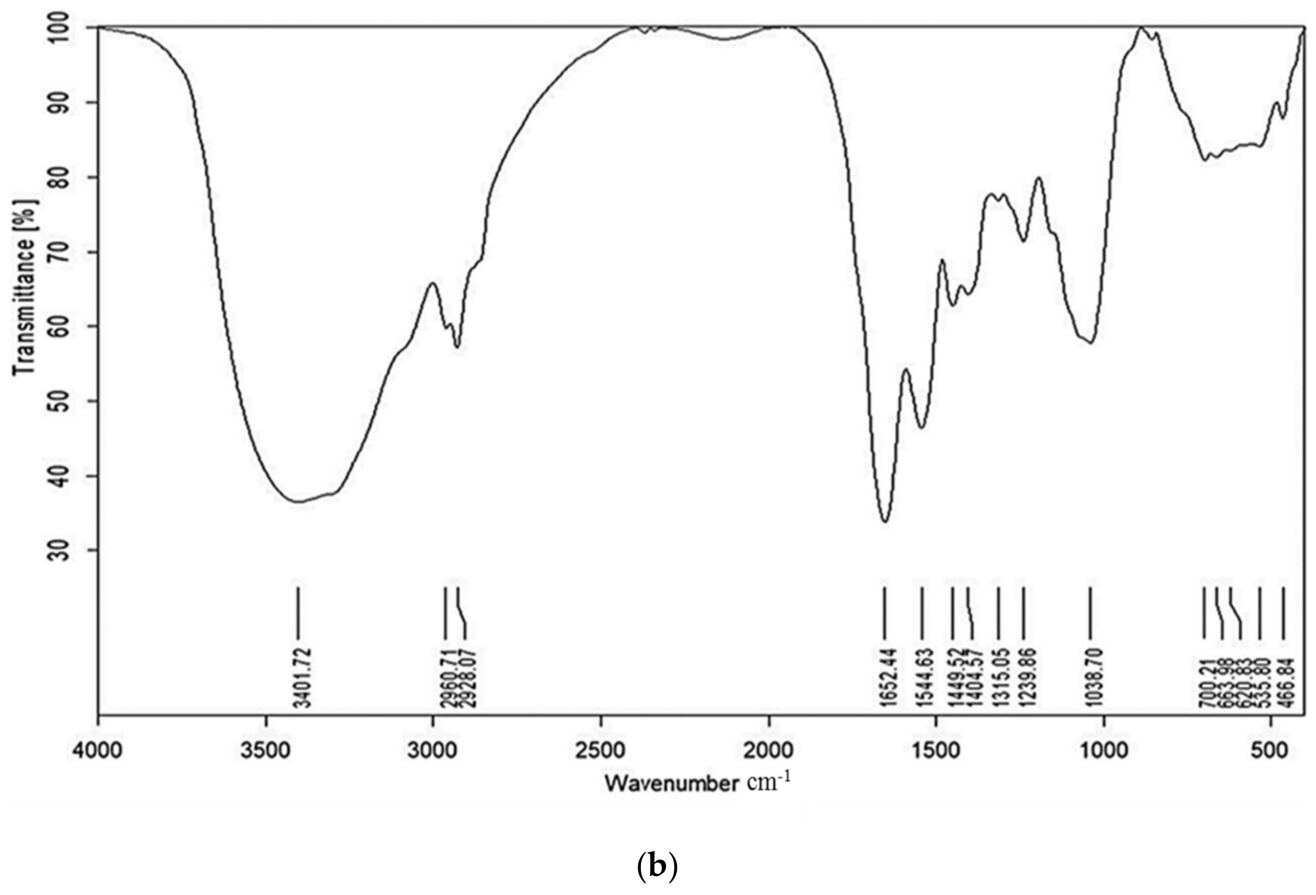
FT-IR spectra of (**a**) *Ulva intestinalis* aqueous extract; (**b**) *Ulva intestinalis* mediated selenium nanoparticles (UISeNPs).

The presence of functional groups in the aqueous extract of the marine alga U. intestinalis was determined using FT-IR analysis (Figure 1a). We noticed that major transmission peaks were observed at 3405.4 cm^−1^, 1097.1 cm^−1^, 2136.9 cm^−1^, 1644.5 cm^−1^, and 779.2 cm^−1^ for U. intestinalis extract, which correspond to N–H of amines, glycosidic bond C–O, aliphatic C–H group, carboxylate group, and C–H alkenes stretching vibration, respectively. Furthermore, other peaks occur at different wavelengths, such as 3377.1 cm^−1^, 2930.1 cm^−1^, 1423.7 cm^−1^, 853.9 cm^−1^, 1202.4 cm^−1^, and 717.1 cm^−1^, that represent the hydroxyl group O–H, the aliphatic C–H group, carboxylate groups, C–O–S, S–O, and C–H alkenes.

The absorbance bands of UISeNPs showed a great similarity with those of algal extract, with some shifting in certain peaks, such as the absorbance peaks of N–H, C–O, carboxylate, and C–H alkenes groups that were shifted into 3401.7, 1038.7, 1652.4, and 700.2, respectively (Figure 1b). Additionally, there is an appearance of three new peaks (663.9, 620.8, and 535.7 cm^−1^), which may be related to the advanced bending vibrations of the Se-O bond.

### 2.2. UISeNPs UV Spectra and Zeta Potential

**Figure 2:**
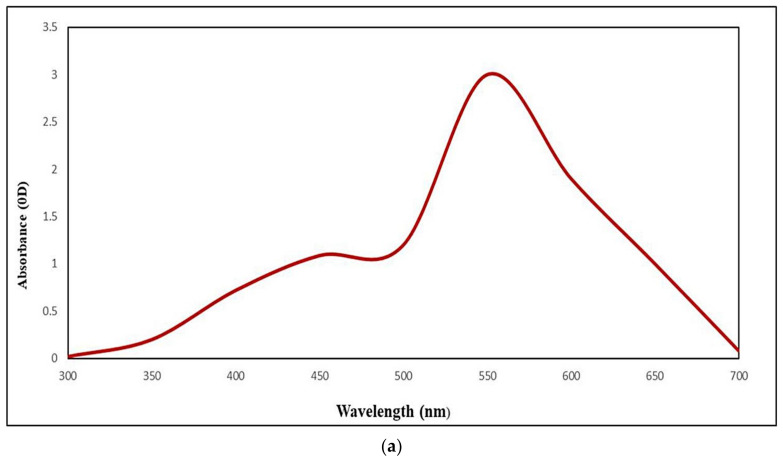

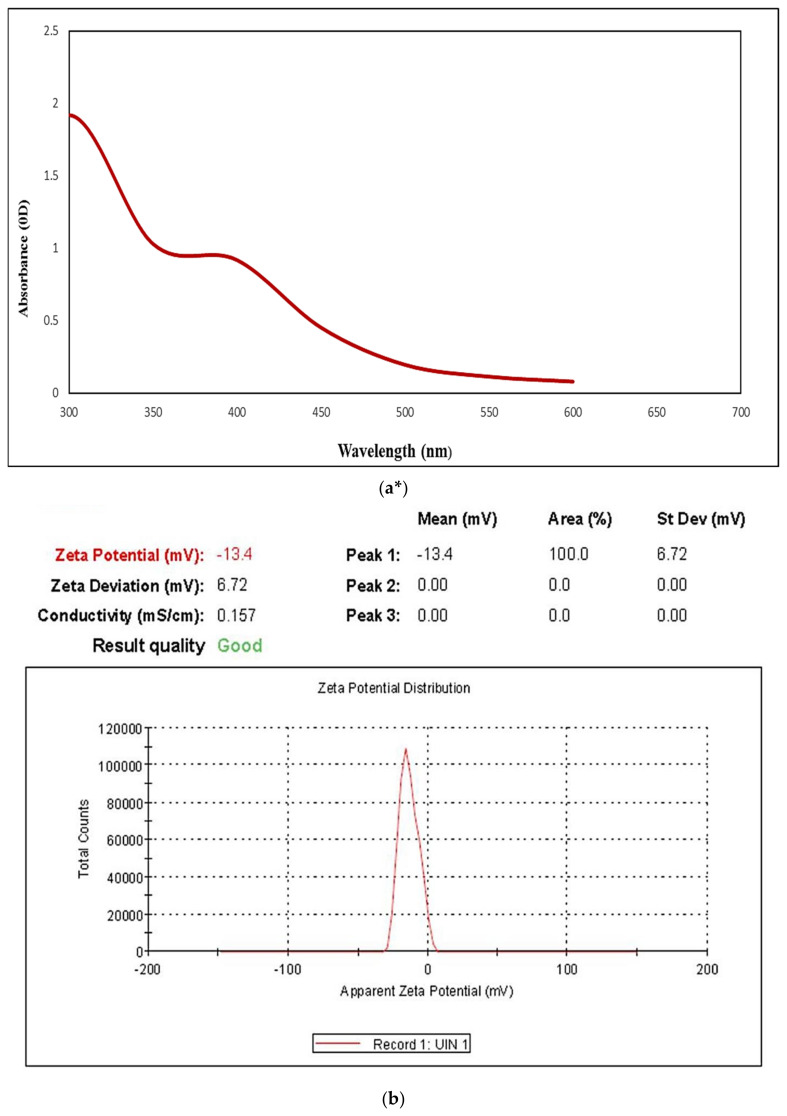

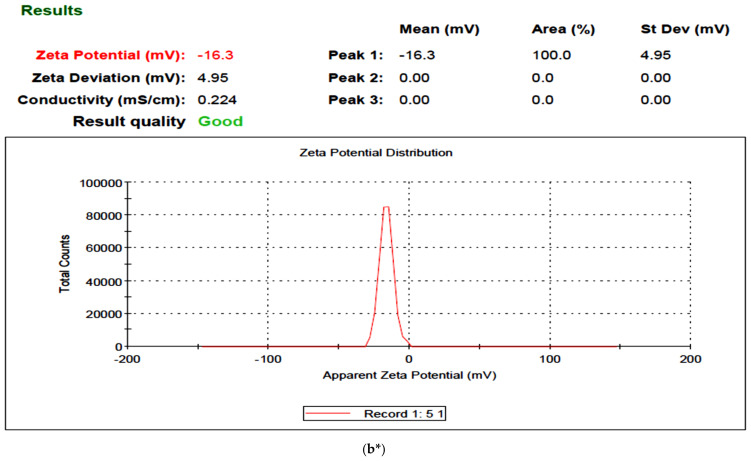
(**a**) The UV-Visible spectrum of *Ulva-intestinalis*-mediated selenium nanoparticles (UISeNPs); (a*) the UV-Visible spectrum of *Ulva intestinalis* aqueous extract; (**b**) zeta potential of UISeNPs; (b*) zeta potential of *Ulva intestinalis* aqueous extract.

UISeNPs synthesis was firstly detected visually by observing the change in color from pale green to ruby red after 48 h, and the absorbance gradually increased from 0.02 to 3.00, with the largest absorbance peak at 550 nm, before it began to decrease again (Figure 2a), whereas the algal extract showed the largest absorbance beak at the range (360–430) nm (Figure 2a*), which indicates the presence of phenolic compounds in the extract, which may be the causative agent for nanoparticles formation [40]. Z potential analysis was employed to detect the stability of the produced NPs; with a value of −13.4 mV (Figure 2b) and a value of −16.3 for algal extract (Figure 2b*), the polydispersity index value was PDI = 0.279.

### 2.3. XRD

**Figure 3:**
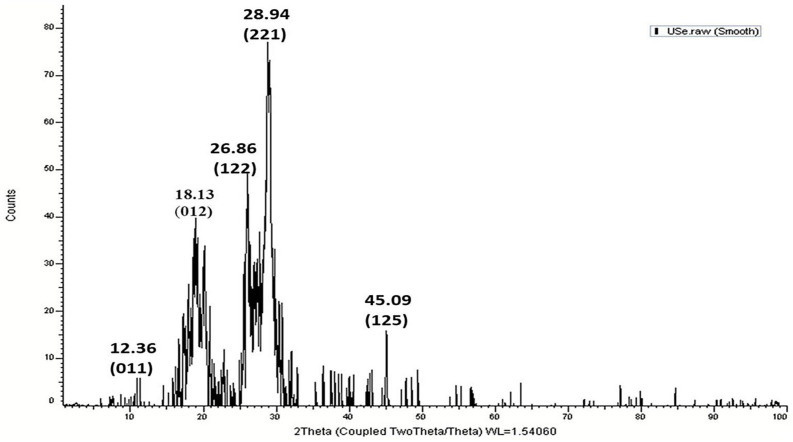
XRD spectra of *Ulva-intestinalis*-mediated selenium nanoparticles (UISeNPs).

XRD analysis was performed to confirm the formation of nanoparticles and determine the crystalline nanoparticle size. As shown in Figure 3, UISeNPs synthesis at 25 °C had intense peaks of 12.36, 18.13, 26.86, 28.94, and 45.09, representing the planes of (011), (012), (122), (221), and (125), respectively. The average size of the crystalline selenium nanoparticle was detected from the XRD peaks by applying the mentioned Scherrer’s equation, and the average size of synthesized NPs was 27.94 nm.

### 2.4. TEM Imaging

**Figure 4:**
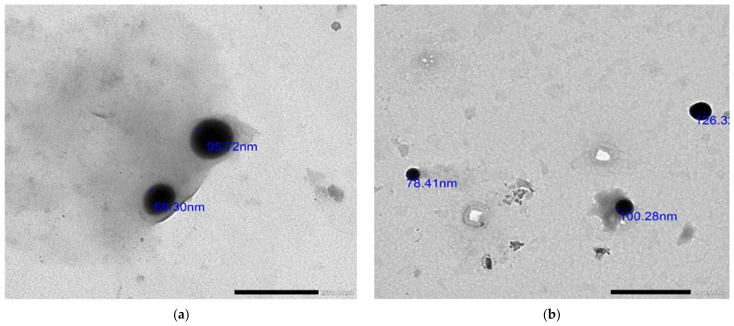
TEM image for *Ulva-intestinalis*-mediated selenium nanoparticles (UISeNPs); (**a**,**b**) different captures 200.0 nm.

The TEM images of the UISeNPs synthesized at 25 °C showed that they are almost spheroidal in shape with a size of 69.3 nm to 126.3 nm (Figure 4).

### 2.5. SEM Imaging

**Figure 5:**
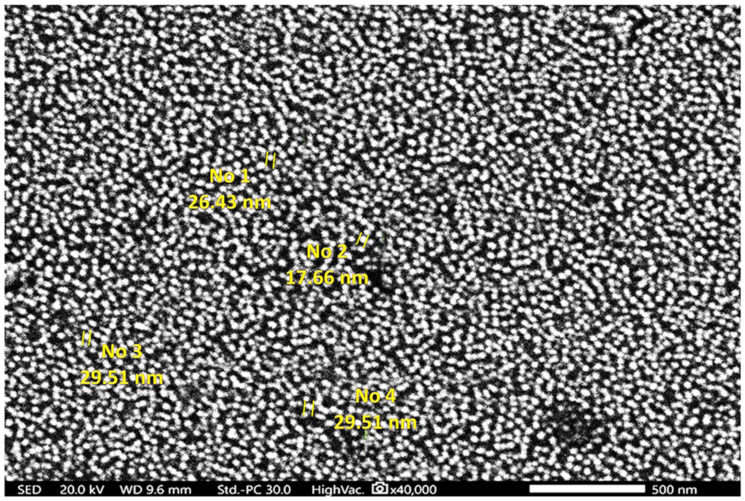
SEM image of *Ulva-intestinalis*-mediated selenium nanoparticles (UISeNPs).

The size of the synthesized UISeNPs is represented in Figure 5. According to the SEM images, UISeNPs had a smooth texture, an oval-to-spherical shape, and the particle size ranged from 17.66 to 29.51 nm.

### 2.6. EDX Analysis

**Figure 6:**
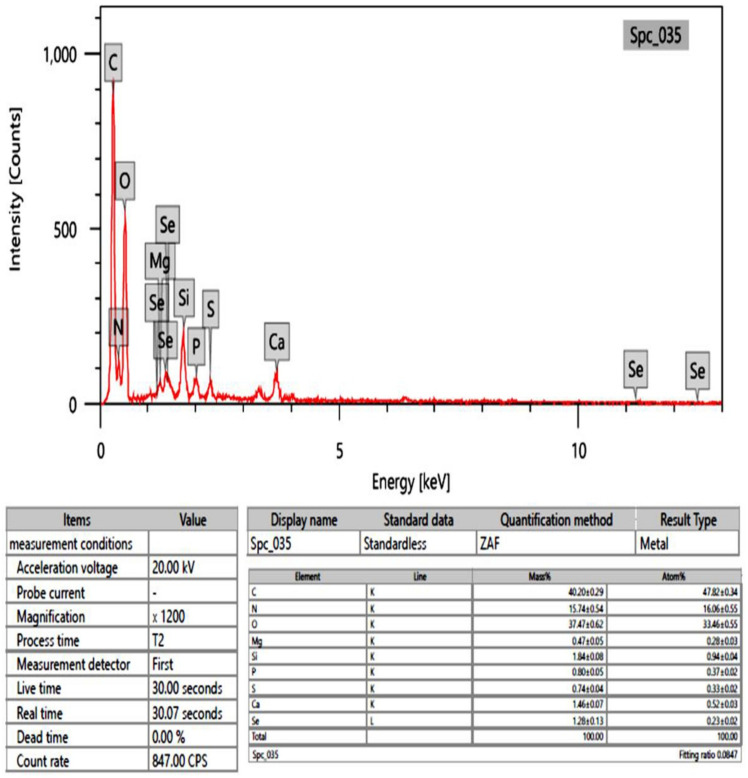
EDX analysis of *Ulva-intestinalis*-mediated selenium nanoparticles (UISeNPs).

Selenium atoms were determined in the elemental composition analysis using EDX (atom % 0.23 ± 0.02; mass % 1.28 ± 0.13) (Figure 6). Our findings revealed the existence of selenium-containing nanostructures together with other EDX peaks such as C, Ca, Mg, N, P, O, S, and Si peaks.

### 2.7. Effect of Treatments on the Weight of Paw Edema

**Figure 7:**
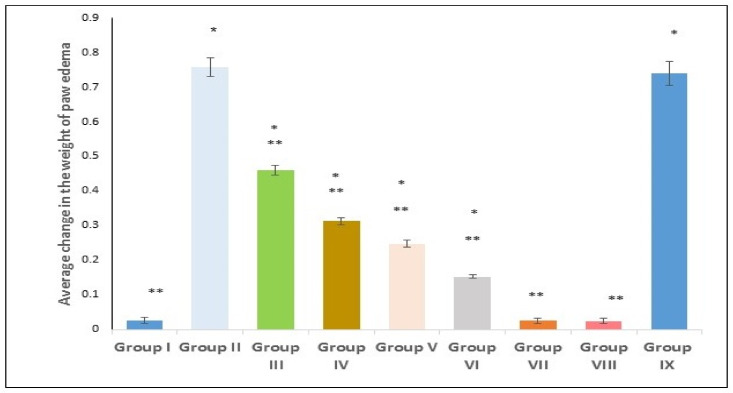
Effect of various treatments on average edema weight changes. Group I (negative control); group II (positive control); group III (pre-treated with free extract 100 mg); group IV (pre-treated with free extract 200 mg); group V (pre-treated with SeNPs loaded with extract 100 mg); group VI (pre-treated with SeNPs loaded with extract 200 mg); group VII (received free extract 200 mg only); group VIII (received placebo SeNPs only); group IX (pre-treated with placebo SeNPs). A single asterisk (*) donates a significant change (*p* < 0.05) vs. the negative control, and double asterisks (**) reveal a significant change (*p* < 0.05) vs. the positive control.

As shown in Figure 7, the average paw edema weight was significantly increased (*p* < 0.00) in Group II (the positive control) compared to the negative control. However, in comparison with group II, groups III, IV, V, and VI revealed a significant decrease in the average paw edema weight (*p* < 0.00; 39.39%, 58.7%, 67.32%, and 79.9% decrease, respectively). Groups treated with the extract loaded on SeNPs (groups V and VI) showed a significant suppression in the average paw edema weight (*p* < 0.00; 20.7%, 51.4% decrease) when compared with group IV (200 mg U. intestinalis extract). Group IX (SeNPs placebo) exhibited no marked change, relative to group II. Data are expressed as mean ± standard deviation (SD).

### 2.8. Effect on Inflammatory and Oxidative Stress Markers

**Figure 8:**
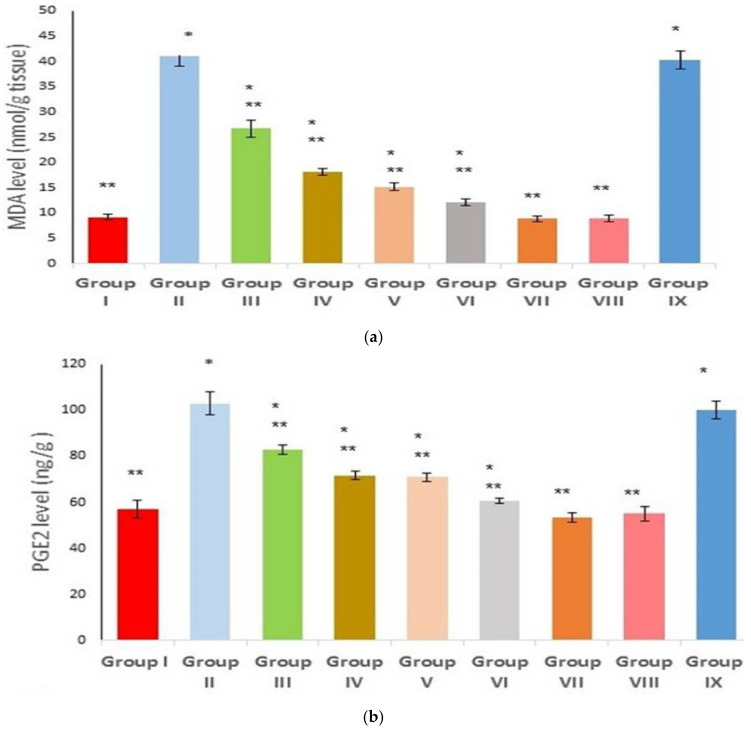
Effect of different treatments on (**a**) MDA levels; (**b**) PGE2 levels. Group I (negative control); group II (positive control); group III (pre-treated with free extract 100 mg); group IV (pre-treated with free extract 200 mg); group V (pre-treated with SeNPs loaded with extract 100 mg); group VI (pre-treated with SeNPs loaded with extract 200 mg); group VII (received free extract 200 mg only); group VIII (received placebo SeNPs only); group IX (pre-treated with placebo SeNPs). A single asterisk (*) represents a significant change (*p* < 0.05) vs. the negative control, and double asterisks (**) means a significant change (*p* < 0.05) vs. the positive control.

As illustrated in Figure 8a, the positive control revealed a substantial increase in the tissue level of MDA (4.4-fold increase, *p* < 0.00). However, the pretreated groups (groups III and IV), in comparison with group II, showed a significant decrease in the MDA levels (*p* < 0.00, 34.8%, 55.8% decrease). It is likely that the groups pretreated with SeNPs loaded with the extract significantly reduced the MDA levels (*p* < 0.00, 62.9%, and 70.2% decreases, respectively), relative to group II (the positive control). There is no significant difference between group II and group IX.

The effect of various concentrations on the tissue content of PGE2 was explored. The positive control group exhibited markedly increased PGE2 levels, relative to the negative control (Figure 8b). By contrast, groups pretreated with the free extract (groups III and IV) revealed a significant decrease (*p* < 0.00) in the MDA content, with percentages of 19.5% and 30.29%, respectively. In comparison with the positive control group (group II), groups V and VI (where rats were pretreated with the extract loaded on SeNPs) showed a substantial decrease in the tissue MDA, with percentages of 31.1% and 41.2%, respectively. Data are expressed as mean ± standard deviation (SD).

### 2.9. Effect on IL-6, Keap 1, Nrf2, and HO-1 Gene Expression

**Figure 9:**
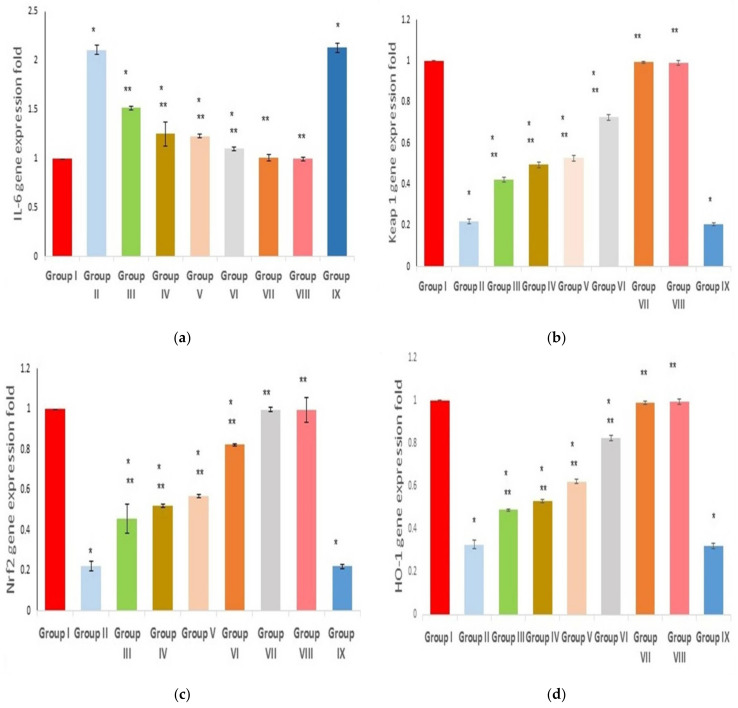
Effect of different treatments on (**a**) IL-6; (**b**) Keap1; (**c**) Nrf2; (**d**) HO-1 gene expressions. Group I (negative control); group II (positive control); group III (pre-treated with free extract 100 mg); group IV (pre-treated with free extract 200 mg); group V (pre-treated with SeNPs loaded with extract 100 mg); group VI (pre-treated with SeNPs loaded with extract 200 mg); group VII (received free extract 200 mg only); group VIII (received placebo SeNPs only); group IX (pre-treated with placebo SeNPs). A single asterisk (*) means a significant change (*p* < 0.05) vs. the negative control, and double asterisks (**) represent a significant change (*p* < 0.05) vs. the positive control.

The effect of Ulva intestinalis extract on IL-6 gene expression was investigated. The positive control group (group II) exhibited an enhancement in IL-6 gene expression (2.11-fold increase, *p* < 0.00), relative to the negative control group, as expressed in Figure 9a. By contrast, the pretreated groups with free U. intestinalis extract (groups III and IV) significantly suppressed IL-6 expression (*p* < 0.00; 27.9% and 40.8% decreases, respectively) when compared with group 2. Furthermore, groups V and VI showed a profound decrease (*p* < 0.00) in IL-6 gene expression (41.7% and 47.4% decrease, respectively), relative to the positive control group II.

As shown in Figure 9b, group II showed a remarkable decrease in Keap1 gene expression (*p* < 0.00; 78% decrease). By contrast, compared to group II, groups III and IV significantly increased the gene expression of Keap1 (*p* < 0.00; 1.9 and 2.27 fold increases, respectively). In comparison with group II, groups VI and 6 revealed a remarkable increase with a fold increase of 2.4 and 3.32, respectively.

Concerning Nrf2 gene expression, group II presented a remarkable decrease (*p* < 0.00; 78% decrease) in comparison to the negative control group (group I). In groups III and IV, Nrf2 expression was significantly increased (*p* < 0.00; 2.09- and 2.36-fold increase) compared with group II. Additionally, the gene expression was remarkably increased in groups V and VI in comparison with group II, with a fold increase of 2.59 and 3.7, respectively (Figure 9c).

Regarding the HO-1 gene expression, group II revealed a profound suppression (*p* < 0.00; 67% decrease) when compared with group I. Wereas groups pretreated with free extract (III and IV) exhibited a significant rise (*p* < 0.00; 1.48 and 1.61 fold increase, relative to group II), HO-1 gene expression was significantly increased in groups V and VI, compared with group II, with a fold increase of 1.87 and 2.84, respectively (Figure 9d). Data are expressed as mean ± standard deviation (SD).

### 2.10. Effect on Keap 1, Nrf2, and HO-1 Protein Level

**Figure 10:**
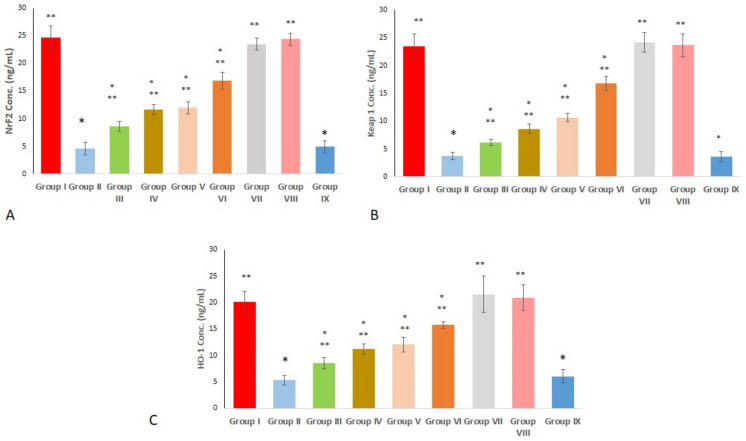
Effect of different treatments on (**A**) Nrf2; (**B**) Keap1; (**C**) HO-1 protein levels. Group I (negative control); group II (positive control); group III (pre-treated with free extract 100 mg); group IV (pre-treated with free extract 200 mg); group V (pre-treated with SeNPs loaded with extract 100 mg); group VI (pre-treated with SeNPs loaded with extract 200 mg); group VII (received free extract 200 mg only); group VIII (received placebo SeNPs only); group IX (pre-treated with placebo SeNPs). A single asterisk (*) means a significant change (*p* < 0.05) vs. the negative control, and double asterisks (**) represent a significant change (*p* < 0.05) vs. the positive control.

Concerning Nrf2 protein levels, group II presented a remarkable decrease (*p* < 0.00; 81.5% decrease) in comparison to the negative control group (group I). In groups III and IV, the Nrf2 level was significantly increased (*p* < 0.00; 1.87- and 2.5-fold increase) compared with group II. Additionally, the protein level was remarkably increased in groups V and VI in comparison with group II, with a fold increase of 2.6 and 3.6, respectively (Figure 10A).

As shown in Figure 10B, group II showed a remarkable decrease in Keap1 protein level (*p* < 0.00; 80.5% decrease). By contrast, compared to group II, groups III and IV significantly increased Keap1 (*p* < 0.00; 1.63- and 2.3-fold increases, respectively). In comparison with group II, groups VI and 6 revealed a remarkable increase, with a fold increase of 2.8 and 4.5, respectively.

Regarding the HO-1 protein, group II revealed a profound suppression (*p* < 0.00; 73.5% decrease) when compared with group I. Whereas groups pretreated with free extract (III and IV) exhibited a significant rise (*p* < 0.00; 1.61 and 2.1 fold increase, relative to group II), HO-1 gene expression was significantly increased in groups V and VI, compared with group II, with a fold increase of 2.3 and 2.97, respectively (Figure 9d). Data are expressed as mean ± standard deviation (SD).

### 2.11. Histopathological Examination

**Figure 11:**
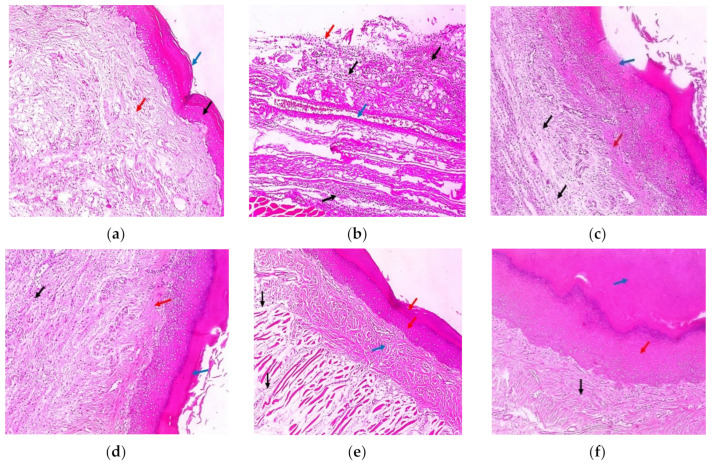

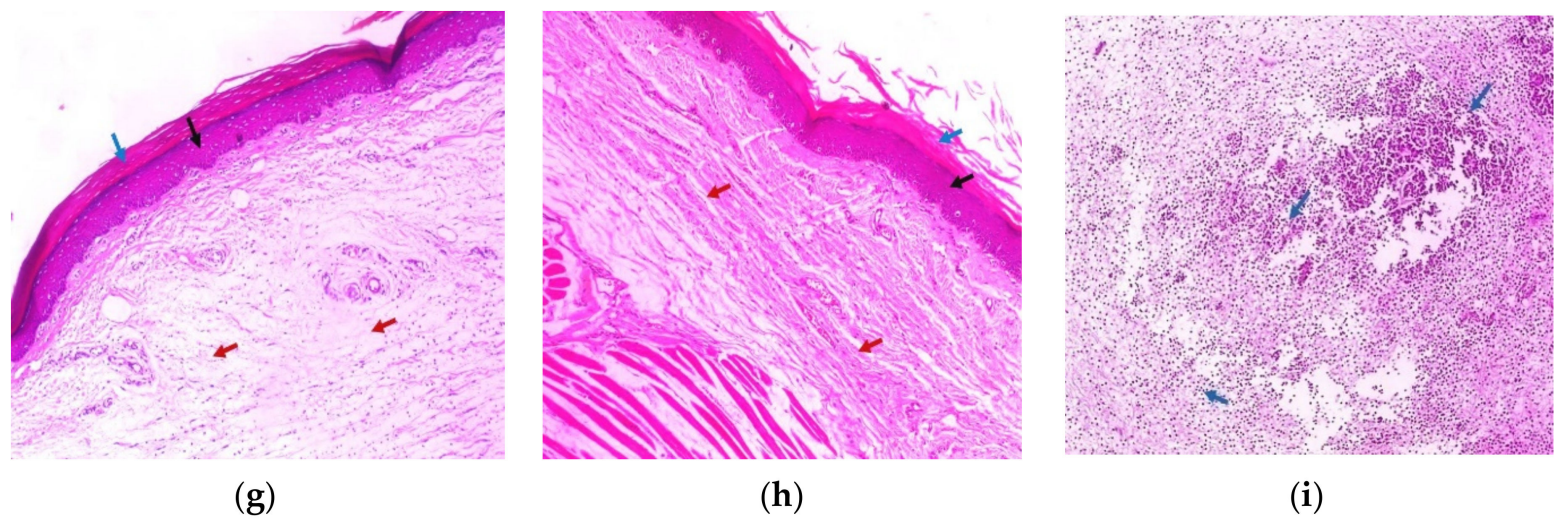
Hematoxylin and eosin (H&E-stained paw sections) (×100). (**a**) Group I (negative control); (**b**) group II (positive control); (**c**) group III (pre-treatment with free extract 100 mg); (**d**) group IV (pre-treatment with free extract 200 mg); (**e**) group V (pre-treated with SeNPs loaded with extract 100 mg); (**f**) group VI (pre-treatment with SeNPs loaded with extract 200 mg); (**g**) group VII (received free extract 200 mg only); (**h**) group VIII (received placebo SeNPs only); (**i**) group IX (pre-treated with placebo SeNPs).

The skin sections from the various groups after being stained with hematoxylin and eosin (H&E) are presented in Figure 11. Group I (negative control) demonstrated normal skin with an epidermis of average thickness (black arrow) lined with thick keratin (blue arrow) and a normal dermis (red arrow). Group II (positive control) shows surface ulceration (red arrow) with underlying granulation tissue containing congested blood vessels (blue arrow) and infiltration by severe chronic inflammatory cells (black arrows). The pre-treatment with free extract 100 mg and 200 mg revealed moderate dermal inflammation (black arrows), thickened epidermis with partial keratosis (blue arrow), and underlying mild collagenosis (red arrow), such as in group III; and mild dermal inflammation (black arrow), thickened epidermis, excessive keratosis (blue arrow), and mild collagenosis (red arrow), such as in group IV. Few dermal inflammations (black arrows), thickened epidermis, moderate keratosis (red arrows), and underlying moderate collagenosis (blue arrow) were observed in group V (pre-treated with SeNPs loaded with extract 100 mg). Moreover, no inflammation (black arrow), thickened epidermis (red arrow), excessive keratosis (blue arrow), and underlying excessive collagenosis (black arrow) were detected in group VI. Normal skin, an average-thick epidermis (black arrow), thick keratin (blue arrow), and an underlying normal dermis (red arrows) were demonstrated in group VI. The rats that received placebo SeNPs only showed normal skin, an average-thickness epidermis (black arrow), thick keratin (blue arrow), and an underlying normal dermis (red arrows) (Group VIII). Group IX (pre-treated with placebo SeNPs) showed skin ulceration with dermal infiltration and heavy chronic inflammatory cells (red arrows) surrounding the nerve bundle (black arrow).

### 2.12. Immunohistochemical Examination

**Figure 12:**
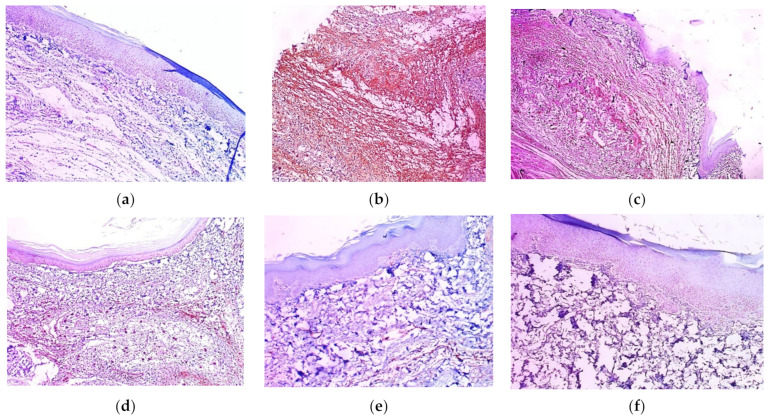

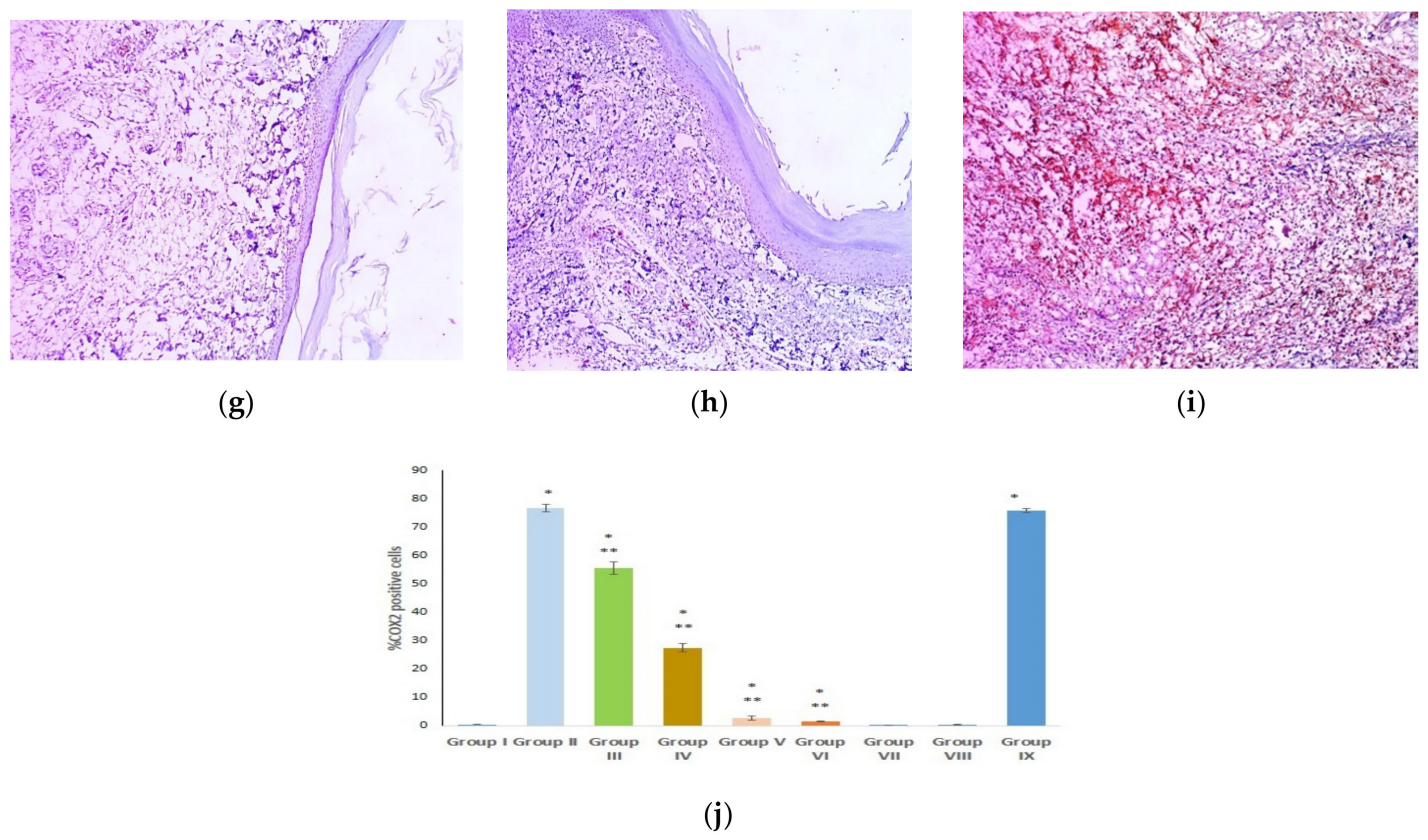
COX2 immuno-stained paw skin sections (×100). (**a**) Group I (negative control); (**b**) group II (positive control); (**c**) group III (pre-treated with free extract 100 mg); (**d**) group IV (pre-treated with free extract 200 mg); (**e**) group V (pre-treated with SeNPs loaded with extract 100 mg); (**f**) group VI (pre-treated with SeNPs loaded with extract 200 mg); (**g**) group VII (received free extract 200 mg only); (**h**) group VIII (received placebo SeNPs only); (**i**) group IX (pre-treated with placebo SeNPs); (**j**) %COX2-positive cells (cell count/1000 cell). A single asterisk (*) represents a significant change (*p* < 0.05) vs. the negative control, and double asterisks (**) means a significant change (*p* < 0.05) vs. the positive control.

The immuno-staining for COX-2 in the tissues of the various experimental groups is demonstrated in Figure 12. Group I (negative control) showed a negative COX-2 expression score of 0. However, group II (positive control) showed marked COX-2 expression, with a score of 4. The pre-treatment with free extract 100 mg showed strong COX-2 expression, with a score of 3 (group III), whereas free extract 200 mg showed moderate COX-2 expression, with a score of 2 (group IV). The COX-2 expression was weak in group V (score 1) and group VI (score 1) and negative (score 0) in groups VII and VIII. The pre-treatment with placebo SeNPs showed strong COX-2 expression, with a score of 4 (group IX).

**Figure 13:**
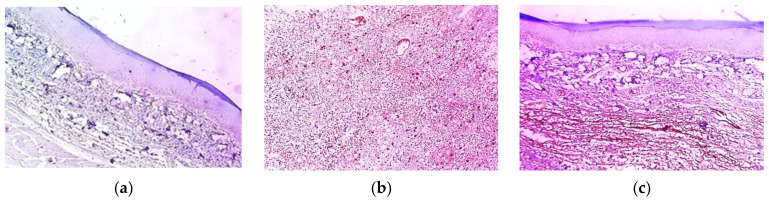

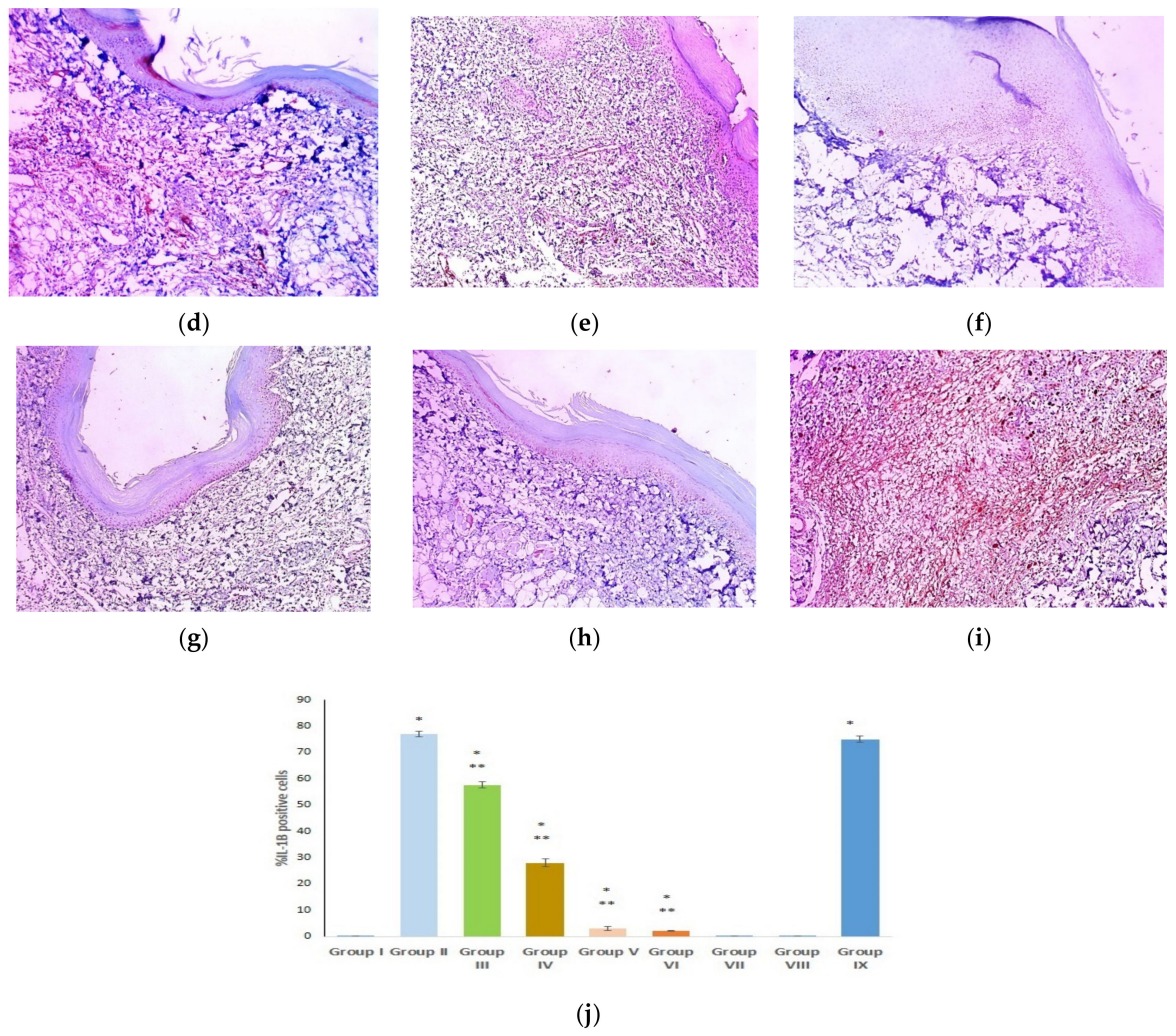
IL-1β immuno-stained paw skin sections (×100). (**a**) Group I (negative control); (**b**) group II (positive control); (**c**) group III (pre-treated with free extract 100 mg); (**d**) group IV (pre-treated with free extract 200 mg); (**e**) group V (pre-treated with SeNPs loaded with extract 100 mg); (**f**) group VI (pre-treated with SeNPs loaded with extract 200 mg); (**g**) group VII (received free extract 200 mg only); (**h**) group VIII (received placebo SeNPs only); (**i**) group IX (pre-treated with placebo SeNPs); (**j**) %IL-1B-positive cells (cell count/1000 cell). A single asterisk (*) represents a significant change (*p* < 0.05) vs. the negative control, and double asterisks (**) means a significant change (*p* < 0.05) vs. the positive control.

The immuno-staining for IL-1β in the tissues of the various experimental groups is demonstrated in Figure 13. A negative IL-1β expression score of 0 was detected in group I (negative control), group VII (received free extract 200 mg only), and group VIII (received placebo SeNPs only), whereas marked IL-1β expression, with a score of 4, was observed in group II (positive control) and group IX (pre-treated with placebo SeNPs). A strong IL-1β expression, with a score of 3, was obvious in group III (pre-treated with free extract 100 mg). The pre-treatment with free extract 200 mg showed moderate IL-1β expression, with a score of 2. Moreover, group V (pre-treated with SeNPs loaded with extract 100 mg) and group VI (pre-treated with SeNPs loaded with extract 200 mg) showed weak IL-1β expression, with a score of 1.

## 3. Discussion

The complex biomolecular constitution of *Ulva intestinalis* (UI) extract and prior studies on SeNPs biological activity motivated us to examine the anti-inflammatory and cytoprotective effects of UI extract and UISeNPs. The final morphology of NPs is determined by their capping, which involves molecules adhering to their surface. This prevents NPs from overgrowing and re-aggregating [41]. The Fourier transform infrared technique is a crucial tool for determining the attachment of functional groups between metals and bioactive molecules. It is used to analyze the chemical constituents of the nanoparticles’ surfaces and identify the bioactive molecules that cap and effectively stabilize the nanoparticles [42]. The absorbance bands at 3405 cm^−1^ and 3377 cm^−1^ were assigned to the N–H and O–H stretching vibrations of amines and hydroxyl groups, whereas the band at 2930 cm^−1^ to 2136 cm^−1^ can be assigned to the aliphatic C–H group stretching vibrations. The signals at 1097 cm^−1^ can be related to the glycosidic bond C-O stretching vibrations. The carboxylate groups exhibit two broad bands at 1644 cm^−1^ and a symmetric stretching band at 1423 cm^−1^ [17,43]. The presence of the C–O–S bending vibration at 853 cm^−1^ and the S–O stretching vibration at 1202 cm^−1^ are related to sulfated esters, confirming the presence of sulfate groups in the polysaccharide structure [44]. The peaks at 779 cm^−1^ and 717 cm^−1^ are assigned to the C–H alkenes stretch [45]. In light of these results, we deduced that *U. intestinalis* included a variety of phytochemicals, including phenolic compounds, lipids, alcohol, proteins, fatty acids, alkaloids, and other constituents with varied biological properties. Moreover, these phytochemicals may have aided the conversion of sodium selenite to SeNAPs [46,47,48]. At 691 cm^−1^ and 4684 cm^−1^, additional bending vibrations of the Se-O bond are observed [22]. These bands were all assigned based on earlier reporting [17,42,43,44,45].

An earlier study that used the marine alga *Ulva lactuca* to produce SeNPs reported comparable SPR vibrations. Size, shape, the dielectric constant of the metal and the surrounding medium, and the frequency and width of the surface plasm on absorption are all affected by the metal nanoparticles [39], as they detected the selenium nanoparticles at 610 nm. According to Z potential analysis, UISeNPS creates a stable colloidal suspension with particles that have a negative electrostatic surface charge, with values of −13.4 mV [48]. Since the extract may be employed as a reducing and capping agent, no further compounds were required. This has been confirmed by estimating the polydispersity index value (PDI = 0.279). A PDI value of less than 0.5 is regarded as appropriate for drug delivery because it indicates how homogeneous the nanoparticles are. The lower the PDI, the more homogeneous nanoparticles are formed. It is a marker of particle aggregation because a higher value indicates a polydisperse system [45].

Regarding XRD results, the average size of nanoparticles was determined from the XRD peaks using Scherrer’s equation D = 0.94λ/β cosθ [49]. XRD peaks were consistent with reference to the unit cell of the face-centered cubic (FCC) structure of metallic selenium (Joint Committee for Powder Diffraction Standards, JCPDS File). The estimated “D” of UISeNPs using Scherrer’s equation (27.94 nm) is in agreement with TEM and SEM results. Moreover, the XRD findings agree with previous studies [50,51].

Further characterization of synthesized selenium nanoparticles was performed to detect the size and shape of the SeNPs with the help of transmission electron microscopy [52]. Our findings were in agreement with Vikneshan et al., 2020, who synthesized SeNPS using *Ulva intestinalis* and found that the shape of SeNPS using TEM was smooth, spherical, and dispersed homogeneously [47]. The detected characters of UISeNPs from SEM were complementary for and confirmed for those obtained from TEM imaging; SEM also showed a crowded background for the synthesized nanoparticles, which improves the capping of nanoparticles by the algal extract [22,39]. EDX analysis confirms the formation of SeO particles through the presence of O and Se in atom percepts 33.46 ± 0.55 and 0.23 ± 0.02, respectively. A further indication that the selenium salt precipitates were mixed precipitates is the presence of additional elements, such as C, Ca, Mg, N, P, O, S, and Si, along with the selenium.

Inflammation is a process that contributes to disease aggravation through the production of different mediators [53]. For the purpose of studying the possible anti-inflammatory effects of compounds, some animal models were documented. One of the most common models is carrageenan-induced paw edema. Carrageenan is a well-known chemical that possesses the ability to propagate the production of several pro-inflammatory and inflammatory mediators [54].

The most outstanding hallmark of the local inflammatory response is edema, which follows the interstitial fluid accumulation in the tissues [55]. The cells of the tissue with edema showed a poor metabolic rate, which could be explained by scant nutrients and oxygen, and resulted from the increased distance of diffusion produced by accumulated fluid [56]. In the present work, the carrageenan-induced inflammation group (positive control) showed marked paw edema, which was consistent with the histopathological examination that revealed infiltration of severe inflammatory cells and blood vessel congestion. In addition, the pretreated groups exhibited a significant reduction in the paw edema weight in comparison to the positive control group, which was supported by the enhanced histopathological findings and the decreased inflammatory cell infiltration. Furthermore, groups treated with SeNPs loaded with *Ulva intestinalis* showed a substantial decrease in comparison with other treated groups, with the higher dose showing the upper hand in revealing such an effect.

In carrageenan-induced inflammation, various pathways are activated, including the COX2 pathway, which, subsequently, increases the production of the inflammatory mediator PGE2 [57]. In the present study, the carrageenan group showed a significant increase in PGE2 expression and a score of 4 for COX2 immune staining. However, the pretreated groups (either with the free or SeNPs decorated with the extract) revealed a significant decrease in PGE2 content and improved the COX2 scoring when compared to the positive control group. Earlier reports were in line with the current data, where *U. intestinalis* showed an anti-inflammatory effect in a rheumatoid arthritis model by decreasing the inflammatory cell infiltration and, subsequently, the accompanied PGE2 [58,59]. Interestingly, the SeNPs loaded with *U. intestinalis* reflected a substantial effect compared to the other treated groups. An effect might be attributed to the ability of nanoparticles to enhance drug bioavailability and efficacy.

Herein, the *Ulva intestinalis* pretreated groups (free or SeNPs loaded) suppressed the score of IL-1β and significantly decreased the gene expression of IL-6. Likely, other reports support these findings: where the extract proved its potential to lessen the production of IL-1β [59], IL-1β was reported to induce COX2 expression and, hence, PGE2 production, where they act side by side to worsen the inflammatory response [60].

Malondialdehyde, as a product of oxidative stress, is involved in vast inflammatory events such as arthritis, gastric ulcer, and carrageenan-induced hind paw edema [61,62,63]. It was found that MDA is closely correlated with the COX2 pathway, as well as PGE2-mediated oxidative stress and its related events [64]. Additionally, IL-1β is directly correlated to the imbalance between antioxidant and oxidant status in favor of oxidative stress and eventually MDA production [65]. Herein, pre-treating carrageenan-injected rats with the free or nanoparticle-loaded extract exhibited a profound decrease in MDA tissue content, with the nanoparticle-decorated formula showing a stronger outcome.

The present study is the first of its kind to assess the possible role of Keap1, Nrf2, and the HO-1 pathway in mediating the protective effect of *Ulva intestinalis* extract. Herein, the pretreatment with the extract, in both its free and nanoparticle-loaded forms, remarkably elevated the gene expression of Nrf2, Keap1, and HO-1. It is worth mentioning that the 200 mg of *Ulva* extract embedded with the SeNPs showed the most significant results among all treated groups. Previously, researchers reported Nrf2/HO-1 as one of the endogenous pathways that alleviate oxidative stress/ROS-associated damage [10,65]. Nrf2 acts on its response element on the DNA to increase the expression and activation of HO-1, thus exerting its anti-inflammatory and antioxidant effects. Different natural agents have proven their potential for activating this pathway, such as tea polyphenols and baicalein extract [66,67].

## 4. Materials and Methods

### 4.1. Ulva Intestinalis Collection, Processing, and Aqueous Extract Preparation

*Ulva intestinalis* was obtained from the rocks of Abu Quir Bay, Alexandria, Egypt. They were directly transported to the laboratory in containers full of seawater to retain the moisture in the algal samples. Algae identification was performed by Prof. Dr. Mohamed Saad Abd El-Kareem, Professor of Phycology, Botany and Microbiology Department, Faculty of Science, Alexandria University, Egypt, as presented in the literature and following the methods of Aleem (1978) [68], Aleem (1993) [69], and Lipkin and Silva (2002) [70], and confirmed using the Algae Base website (M.D. Guiry in Guiry 2016) [71].

Algal samples were properly cleaned with water and filtered seawater to remove foreign objects, and then shade-dried for five days before being oven-dried at 60 °C to achieve a constant weight. Finally, the material was ground into tiny particles using an electric mixer and stored in a dark place at room temperature for subsequent testing. A mixture of 10 grams of algal powder and 100 mL of water was boiled for 30 min with stirring. The supernatant that was collected using a 10,000-rpm centrifuge was added as a reducing agent for Na_2_SeO_4_ [72].

### 4.2. Preparation of Ulva-Intestinalis-Mediated Selenium Nanoparticles (UISeNPs)

*Ulva-intestinalis*-mediated selenium nanoparticles (UISeNPs) were synthesized as reported by El-Saied et al., 2021 [73], with slight modifications, e.g., the drop-wise addition of freshly prepared *U. intestinalis* extract solution was done to 1 mM Na_2_SeO_4_. The mixtures were kept under 10 min of vigorous stirring for complete homogeneity, and then kept in light static conditions at 25 ± 2 °C until the color of the sodium selenate solution converted to orange-red, demonstrating the formation of phycosynthesized UISeNPs, which were detected by measuring the absorbance at 300–800 nm. The algal extract was detected for its component by measuring the absorbance at 200–450 nm [40] using UV spectroscopy (Thermo Scientific Evolution TM 300, Thermo Fisher Scientific, Waltham, MA, USA) [73]. The mixture was centrifuged for 30 min at 10,000 rpm. The NPs were then washed with water, rinsed with pure ethanol, baked at 50 °C, and preserved in a tightly closed container [39].

### 4.3. Characterization of Phycosynthesized SeNPs

#### 4.3.1. Fourier Transform Infrared Spectroscopy (FT-IR)

The algal extract and UISeNPs were analyzed using FT-IR in attenuated total reflectance (ATR) mode on a PerkinElmer System 2000 analyzer to determine the adsorption of functional groups. The specimens were prepared using the KBr pellet technique [41]. The IR-transparent KBr was blended with 150 parts with the dried algal sample. The blend was subsequently refined to an adequate, uniform powder that was pressed at high pressure (5 to 6 bar) for 5 min to form a disc pellet with a thickness of evenly 1 mm. For precise measurements, the pellets with low quality were excluded.

The average % transmittance values were charted against wavelength (4000–400 cm^−1^) after the spectra for each specimen were recorded three times [41]. In order to define the functional groups, the FT-IR spectrum was examined and related to the absorption band values.

#### 4.3.2. X-ray Diffraction (XRD) Analysis

In order to confirm the crystallinity and phase composition of the NPs, XRD analysis was conducted. The 2.2 KW Cu anode radiation used in this investigation was measured using an XRD 6000 detector (Shimadzu Corp., Kyoto, Japan) at 30 kV and 10 mA, where k = 1.54184.

The average size of the NPs (D) was calculated using Scherrer’s equation (D = λk/βcosθ). Here, k is a constant that was almost equal to 0.9; the X-ray wavelength (λ) is 1.54060; β is the peak width induced by the size effect measured in radians; and θ, which was calculated using the formula (θ1 − θ2) π/180, is the Bragg diffraction angle. To confirm the particle size of the material, the line width of the (221) reflection in XRD was specifically employed [18].

#### 4.3.3. Transmission Electron Microscopy (TEM)

The size and morphology of UISeNPs were investigated using TEM (JEM 2100, JEOL, Ltd., Tokyo, Japan) at an accelerating voltage of 200 kV. Briefly, nanoparticles (at a concentration of 1 mg/mL) were suspended in deionized water. The mixture was subsequently sonicated (Branson Sonifier 250, Branson Ultrasonics Corp., Brookfield, CT, USA) for ten minutes until developing uniform suspension. In order to measure NP size, the suspension was diluted twenty times, and a drop of diluted suspension was placed on copper grids coated with carbon and vacuum-dried for 30 min. Finally, the sample was then examined using a transmission electron microscope [18].

#### 4.3.4. Scanning Electron Microscopy (SEM) and Energy-Dispersive X-ray (EDX)

Using a SEM (JSM 6490 LV, JEOL, Ltd., Tokyo, Japan), UISeNPs were examined at operating parameters of 0–15 kV, 45 nA, and counting the duration of 60 s. The nanoparticles (1 mg) were dispersed in one millimeter of deionized water, followed by sonication in order to obtain a homogenous mixture. Further dilution was required, followed by placing a drop of the diluted suspension on a glass holder. After drying, gold coating of the sample was performed, and NPs were examined to determine their form and diameters [18].

The energy-dispersive X-ray (EDX) analysis was performed using a JSM 6490 LV (JEOL) instrument operated at 200 kV in order to determine the elemental compositions of the particles. Images were captured at magnifications of 50 and 100 kV.

#### 4.3.5. Zeta Potential (ζ) and Polydispersity Index (PDI)

At 25 °C, Zeta Plus (Brookhaven, New York, NY, USA) measured the size, polydispersity index (PDI), and zeta potential of each sample. At a final concentration of 100 µg/mL, the values of the zeta potential were determined in the nanoparticle suspension with a continuous measuring of pH prior to each individual measurement. Based on the particle dispersion, the PDI was determined. Triplicate analyses of all samples were performed [18].

### 4.4. Experimental Model for Inflammation Induction

Inflammation was induced by injecting the sub-planter right hind paw with 0.2 mL of subcutaneous (SC) injection of freshly prepared 1% carrageenan solution in normal. To preserve control, the left hind paw was left uninjected [74].

#### 4.4.1. Animals

Fifty-four male Wistar albino rats were obtained from the animal house located at the Faculty of Veterinary Medicine, Cairo University, Egypt. The animals weighed 170–200 g and were supplied with filtered water and standard pellets at 25 ± 2 °C and 12 h-light/dark cycle. The in vivo methods and protocol were accredited by the Research Ethical Committee (Faculty of Pharmacy, Tanta University, Egypt), as aligned with the standard rules of handling and care of the laboratory animals (TR/RE/11/21P-0052)

#### 4.4.2. Experimental Design and Animal Groups

The rats were randomly assigned into nine groups (six rats per group). Group I represented the negative control group and group II was the positive control group. The rats in both groups were orally administered 0.9% saline (10 mL/kg). The rats of groups III, IV, V, and VI orally received 100 and 200 mg/kg of *Ulva intestinalis* extract and 100 and 200 mg/kg of UISeNPs, respectively. Group VII orally received *U. intestinalis* extract and groups VIII and IX received SeNPs placebo. The amount of the extract loaded on SeNPs was equivalent to free *U. intestinalis* given to groups III and IV. The doses of *U. intestinalis* extract were chosen based on the doses mentioned in previous studies [75,76]. After one hour, inflammation was induced by carrageenan injection in rats of all groups except groups I, VII, and VIII. After six hours, the animals were anesthetized with isoflurane, and the euthanasia of rats was performed by cervical dislocation (CD) according to the American Veterinary Medical Association (AVMA) Guidelines for the Euthanasia of Animals (2020 Edition). Both left and right paws were amputated at the same position and weighed [77]. The average edema weight was calculated using the difference between the weights of both paws [78]. The analyst was blinded regarding which rats represented control and treatment groups.

#### 4.4.3. Sample Collection

After weighing the paws, the right paw tissue was taken and divided into two parts. One part was snap-frozen in -80 freezer to determine IL-6, Keap1, HO-1, Nrf2, Malondialdehyde (MDA), and prostaglandin E2 (PGE2). The rest was kept in formalin for further histopathological and immunohistochemical staining of COX2 and IL-1β.

#### 4.4.4. Measurement of Prostaglandin E2 (PGE2)

The PGE2 level was determined in the paw tissues, which were homogenized in ice-cold PBS (1: 9, *v*/*w*), using an Enzyme-Linked Immunosorbent Assay (ELISA) kit (Creative-Biolabs, Shirley, NY, USA) at 450 nm with an ELISA reader (Sunrise, Switzerland). In brief, the extract was added to pre-coated wells, followed by addition of biotin, antibodies labeled with streptavidin-horseradish peroxidase, and chromogens. Then, the absorbance was measured after adding the stop solution.

#### 4.4.5. Measurement of Malondialdehyde (MDA)

The tissue content of MDA was determined using Biodiagnostic kits (Giza, Egypt) at 540 nm, and the steps were conducted as described by the manufacturer protocol. Briefly, the tissues were homogenized in ice-cold PBS to produce a 10% homogenate suspension. After that, supernatants were eliminated, and MDA levels were measured using an ELISA reader. Following the attached formula, the concentrations were calculated.

$$\mathrm{MDA}\;\mathrm{level}\;\left(\mathrm{nmol}/g\;\mathrm{tissues}\right)=\frac{A\;\mathrm{sample}}{A\;\mathrm{standard}}\times \frac{10}{\mathrm{weight}\;\mathrm{of}\;\mathrm{tissues}\;\left(g\right)}$$

#### 4.4.6. Measurement of IL-6, HO-1, Keap1, and Nrf2 Gene Expression

The gene expression of the inflammatory markers IL-6, HO-1, Keap1, and Nrf2 was determined in the paw tissues using quantitative real-time polymerase chain reaction (qRT-PCR). After the reverse transcription of total RNA into DNA, the primers, in the presence of Syber Green Master Mix, were used for the cDNA amplicon amplification step. Target genes were detected using qRT-PCR under the following conditions: 35 cycles of denaturation at 90 °C for 30 s, annealing at 58 °C for 50 s, and elongation at 72 °C for 35 s. In this assay, the GAPDH gene served as the reference gene. The list of primers that were employed is represented in Table 1.

#### 4.4.7. Measurement of HO-1, Keap1, and Nrf2 Protein Level

The protein level of each HO-1, Keap1, and Nrf2 was measured according to the manufacturer protocol mentioned in the ELISA kits with catalog number (MBS764989, MBS7218529, MBS752046), respectively. All kits were purchased from Biosource, Xenia, OH, USA.

### 4.5. Histological and Immuno-Histochemical Examination

The formalin-preserved paw tissue was immersed in paraffin wax, sectioned (with a 5 μm thickness), and stained with hematoxylin and eosin. The stained sections were inspected under a light microscope. For immunohistochemical staining, the COX-2 and IL-1β expressions were determined by immuno-staining the paw tissues via ABclonal Technology kits (Woburn, MA, USA). The results were assigned scores based on the positive staining percentages. These scores are as follows: score 0 denotes the lack of immune-positive cells; score 1 denotes the presence of up to 25% of immune-positive cells; score 2 denotes the presence of cells that have an immune stain of 11–50%; score 3 denotes the presence of cells that have an immunological stain of 51–75%; and score 4 denotes the presence of cells that have an immune stain of 76–100% [82].

### 4.6. Statistical Analysis

The obtained results were plotted as mean ± standard deviation (SD). One-way analysis of variance (ANOVA) was applied to compare distinctive groups, followed by a post hoc test. The significance of the data was defined as *p* < 0.05.

## 5. Conclusions

To our best knowledge, the present study was the first to highlight the effect of Egyptian Ulva intestinalis extract, either in its free form or loaded on SeNPs, on the inflammation induced by carrageenan in the rat paw and the possible role of the Nrf2/Keap1/HO-1 pathway in mediating such an effect. The free extract has proven its anti-inflammatory effect by mitigating the inflammatory mediators and oxidative stress. Furthermore, it exhibited its action by improving the expression of the cytoprotective pathways Keap1/Nrf2/HO-1. Although the formula of the extract loaded on SeNPs also showed anti-inflammatory and cytoprotective effects, it has the advantage of having the upper hand over the free extract, which might be attributed to the enhanced bioavailability of the SeNPs formula, which could be addressed in future research.

## 6. Limitations

This is a preliminary study that focused on the possible anti-inflammatory/antioxidant/cytoprotective role of Egyptian Ulva intestinalis extract. However, further studies will be conducted to further explore more inflammatory, oxidative stress, and antioxidant markers.

## Figures and Tables

**Table 1:** The primer sequences used for qRT-PCR.

Target Gene	Primer Sequence		Reference
Keap1	Forward Reverse	TAAGAGGAACGGAATG ACATCATCTATTCTCT	[79]
Nrf2	Forward Reverse	CTCATCGTAAGCGAACAATGG GCACCGTTCTTAGCG	[79]
HO-1	Forward Reverse	CTATCGTGCTCGCATGAAC CAGCTCCTCAAACAGCTCAA	[80]
IL-6	Forward Reverse	GCCCTTCAGGAACAGCTATGA TGTCAACAACATCAGTCCCAAGA	[81]
GAPDH	Forward Reverse	AGTGCCAGCCTCGTCTCATAG ACTTGCAACTTGCCGTGGGTAG	[79]

## Data Availability

All data generated or analyzed during this study are included in this published article.
